# Exploring parkrun experiences of women aged 35 to 54 in Australia: a qualitative study

**DOI:** 10.1093/heapro/daag081

**Published:** 2026-06-16

**Authors:** Charlotte Benkowitz, Paul O’Halloran, Helen Quirk, Alice Bullas, Steve Haake

**Affiliations:** School of Psychology and Public Health, La Trobe University, Plenty Road, Bundoora, Melbourne, Victoria 3086, Australia; School of Sport and Physical Activity, Sheffield Hallam University, Olympic Legacy Park, 2 Old Hall Road, Sheffield S9 3TU, United Kingdom; Centre for Sport and Social Impact, La Trobe University, Plenty Road, Bundoora, Melbourne, Victoria 3086, Australia; School of Medicine and Population Health, The University of Sheffield, Western Bank Villa, 300-302 Western Bank, Sheffield S10 2TN, United Kingdom; School of Sport and Physical Activity, Sheffield Hallam University, Olympic Legacy Park, 2 Old Hall Road, Sheffield S9 3TU, United Kingdom; School of Sport and Physical Activity, Sheffield Hallam University, Olympic Legacy Park, 2 Old Hall Road, Sheffield S9 3TU, United Kingdom

**Keywords:** community, physical activity, women’s health, parkrun, community events, mass-participation events, community health promotion

## Abstract

To increase global activity levels, the World Health Organization’s Global Action Plan on Physical Activity includes the implementation of mass-participation, community events that provide an opportunity for individuals to be active in their local spaces. One such initiative is parkrun, with free, weekly 5-km run/walk events worldwide and around 500 events with roughly 50 000 participants every Saturday morning in Australia. While women make up half of parkrun participation in Australia, they face different barriers than men to physical activity and have lower activity levels globally. With the aim of informing how community-based physical activity initiatives like parkrun could increase women’s physical activity levels, 29 semistructured interviews were conducted with women aged between 35 and 54 years old who have participated in parkrun. Using reflexive thematic analysis, four themes were developed based on elements that are important to women in their parkrun experience: (1) parkrun can be whatever you want it to be, (2) the parkrun experience is consistent, (3) parkrun creates connections, and (4) parkrun allows growth and change. Based on these themes, and with the context of health promotion theories, some elements that should be considered when aiming to create initiatives to increase women’s activity levels through community events are to have an element of regularity and repetition; to allow for flexibility in attendance; and to create space for social engagement.

Contribution to Health Promotion

- This study explored how parkrun, a community-based initiative, impacts women's experience of physical activity.
- Parkrun enables women to engage in physical activity within their community and through this improves their health.
- Results include parkrun creating a supportive environment and giving participants autonomy over their physical activity and health behaviours. The initiative impacts individuals beyond an increase in physical activity and contributes to an improvement in skills and confidence.
- The findings can inform the design of other community-based initiatives to increase health and physical activity and highlight the potential that community-based initiatives have to reduce barriers to physical activity for women.

## Background

Physical inactivity is of global concern, due to its increasing burden on healthcare systems (Santos *et al*. 2023) and negative health consequences for individuals (Katzmarzyk *et al*. 2022). Inactivity has been causally linked to the burden of Type 2 diabetes, bowel cancer, dementia, and coronary heart disease (Australian Institute of Health and Welfare 2021). Meanwhile, physical activity (PA) is associated with good physical and mental health and an increase in quality of life and wellbeing (Saxena *et al*. 2005, Reiner *et al*. 2013, Warburton and Bredin 2017, Marquez *et al*. 2020). In Australia, 24.9% of men compared with 19.9% of women meet PA guidelines (Australian Bureau of Statistics 2023b). PA guidelines set by the World Health Organization (WHO) state that adults should do at least 150–300 minutes of moderate-intensity aerobic PA or 75–150 minutes of vigorous-intensity aerobic PA (or a combination of the two), as well as at least 2 days of muscle-strengthening exercise (World Health Organization 2022). The Australian Government’s Department of Health National Women’s Health Strategy is in line with these recommendations (Australian Government Department of Health 2018).

Women being less active than men (Edwards and Sackett 2016, Guthold *et al*. 2018) has multiple potential explanations. Women are expected to fulfil various roles over the course of their lives, including employee, wife, mother, and caregiver; and with these expectations rising, PA levels fall (Peng *et al*. 2023). Biological and bodily changes throughout women’s lives, such as puberty, pregnancy, postpartum and menopause bring challenges and require adaptations of the type, regularity, and intensity of PA and adjusting expectations women hold of themselves (Women and Equalities Committee 2024). Returning to exercise after the transition to motherhood is often more challenging than expected, with women having to juggle priorities (Deery *et al*. 2025). Other barriers include: religious and cultural norms; safety concerns about the physical environment and unsafe neighbourhoods; family duties and a lack of social support (Abbasi 2014, Peng *et al*. 2023).

The WHO’s Global Action Plan on Physical Activity (World Health Organization 2013) recommends mass participation events and free activities in open public spaces, such as parkrun, as a potential strategy to increase population-wide PA levels. Parkrun is a free, community-based PA initiative, predominantly organized by volunteers, where participants can run, walk, or volunteer at 5-km events every Saturday morning (parkrun, 2025) and has been found to increase PA and fitness (Grunseit *et al*. 2020). It originated in London, England, in 2004, expanded to Australia in 2011 with now around 500 events and over 50 000 participants weekly in Australia alone. Prior to participation, individuals register online, easing accessibility, and receive a barcode, which gets scanned together with a position token at the end, giving participants their 5-km finishing time.

A longitudinal study with new registrants found parkrun increased PA by an average of 39 minutes weekly, reduced body mass index, increased happiness and decreased perceived stress and finish times, suggesting an increase in fitness at 6 and 12 months follow-up (Stevinson and Hickson 2019). The only randomized controlled trial in parkrun to-date found that, for adolescents aged 13–18, participating in 6–10 parkruns within a 10-week period, improved cardiorespiratory fitness and finish time (on average 5.5 minutes) and brought small beneficial changes to skeletal muscle mass and body fat (Williams *et al*. 2024). A systematic review assessing the outcomes of parkrun participation found that parkrun can promote improvements in fitness, body mass index, PA levels, mood and personal wellbeing (Peterson *et al*. 2022). Volunteering at parkrun has also been linked to physical and mental health benefits and improvements in feeling part of a community, the number of new people participants meet, and an increase in time spend with friends (Haake *et al*. 2022). Qualitative research of 48 participants in the UK identified that the freedom, referring to the inclusivity and accessibility, and the reciprocity of being able to give back to the community are important in an individual’s parkrun experience (Stevinson *et al*. 2015). Facilitators of participation identified through interviews with 10 participants in Australia were the sense of community, attendance incentives and rewards, opportunities for socializing, lack of fees and feeling safe, with barriers relating to outside commitments, a lack of social support and the weather (Sharman *et al*. 2019). Overall, parkrun can increase PA and has positive impacts on health and wellbeing through being a community-based PA initiative.

Parkrun reaches groups that are traditionally underrepresented in organized sport and PA, including those insufficiently active, aged over 35 and women (Grunseit *et al*. 2020). The gender gap in parkrun has been getting narrower, with an increasing proportion of new parkrun participants in Scotland being female (Gilburn 2023). In Australia, 51.1% of runs, walks, and volunteer occasions between 2011 and 2021 were completed by women and girls (Turne 2021). Parkrun seems to offer an opportunity that helps bridge the gender gap in PA.

The aim of this study was to highlight possibilities for increasing PA levels for women through community-based initiatives, using parkrun as an example. The study explored, through interviews with women who participate in parkrun in Australia, parkrun experiences to give insight into why women participate and what they value about parkrun.

## Method

This study was approved by the Ethics Review System at Sheffield Hallam University and the Human Research Ethics Committee at La Trobe University (ER47577148). Twenty-nine women participated in semistructured interviews. All individuals who had participated in at least one parkrun in Australia were contacted to complete a survey on the health and wellbeing impact of parkrun where they could leave their email address if they were willing to participate in a follow-up interview. Women who had left their email address and were between 35 and 54 years old were contacted in randomized order via email to be invited to participate in an online interview and provided with a participant information sheet. If they agreed, a date and time for the interview was arranged. One hundred and sixteen women were contacted, 35 agreed to be interviewed and 29 interviews were conducted. Prior to the interviews, participants completed a consent form.

The interviews were semistructured, as this allowed for flexibility in the order of questions and to deeper explore elements mentioned by participants (Horton *et al*. 2004, Dearnley 2005). Questions explored participants’ parkrun experience and overall PA perceptions. They were influenced by health promotion theories, such as the COM-B model (Michie *et al*. 2011). After introductory questions, questions related to past PA, participants’ PA journey and changes to their PA through parkrun, for example ‘Could you tell me about your parkrun experience?’ and ‘How is participating in parkrun different from other physical activity you do?’ (see Supplementary Table S1 for the full interview guide). All interviews were conducted by the lead researcher (C.B.). They were recorded and automatically transcribed using Microsoft Teams and manually verified. Average length was 44.3 minutes, ranging between 33.5 and 56.3 minutes. Individuals received an AU$40 supermarket voucher as compensation for their time.

This study focussed on women aged between 35 and 54 for several reasons. Firstly, data from the 2022 National Health Survey in Australia suggested that over 80% of women between 35 and 54 do not meet the PA guidelines proposed by the WHO (Australian Bureau of Statistics 2023b). Secondly, internal parkrun data showed that over 50% of women who have participated in parkrun in Australia fall within this age group (see Supplementary Table S2, Supplementary Figure S1, Supplementary Figure S2). Finally, given that women undergo social and biological changes throughout their life, it was deemed important to capture the experience of women in different life stages: in this age range, women commonly give birth and have young children (Australian Bureau of Statistics 2023a) and experience perimenopause and menopause (Gold 2011).

Demographic information was extracted from the previous survey. No participants identified as Aboriginal or Torres Strait Islander. Four participants spoke a language other than English at home. Twelve participants’ fathers and twelve participants’ mothers had been born outside of Australia (nine participants’ parents were both born outside Australia). Five participants were from the least disadvantaged quintile (level 5), 13 participants were from level 4, 5 from level 3, 1 from level 2, and 4 participants were from the most disadvantaged quintile. The mean number of parkruns completed by the sample was 82.1, ranging from 1 to 299. Twenty participants had volunteered in the past, with volunteering occasions ranging from 0 to 120.

The data were analysed using reflexive thematic analysis where, aligned with Big Q qualitative research, the researcher is not seen as removed from the data, but rather the method values researcher subjectivity (Braun and Clarke 2022). Therefore, establishing the position of the researchers regarding the research is important (see reflexivity statement). Consistent with the underlying philosophies of reflexive thematic analysis, data collection ended when information power was reached, evaluating the data’s richness and detail in relation to its ability to answer the research question (Malterud *et al*. 2016) rather than data saturation. Considerations of information power included the broad aim of the study and limited previous interview experience of the lead researcher suggesting a larger sample size, while the specification of female participants and age group pointed to fewer participants. Qualitative parkrun research and reflections on the data with coauthors also guided decisions. Information power was reached after 29 interviews.

Due to the nonpositivist nature of reflexive thematic analysis, no reporting checklist exists (Braun and Clarke 2024a). However, the following sections are reported in line with recommendations (Braun and Clarke 2024b) and the six steps of reflexive thematic analysis were followed (Braun and Clarke 2022). Data familiarization (phase one) involved the lead researcher conducting the interviews, listening back to audio files and verifying the transcripts. Data coding (phase two) was accomplished using NVivo 14, initially semantic (staying close to the language of the participant), becoming more latent (looking for more implicit meaning and allowing for a deeper understanding of participants’ views), as the analysis progressed. Each transcript was coded at least twice by the lead researcher, reviewing and discussing codes with coauthors regularly to ensure uniqueness. One coder is an appropriate method for reflexive thematic analysis (Braun and Clarke 2022), given the method’s subjective and situated nature. After coding, initial theme generation (phase three); theme development and review (phase four); and theme refining, defining and naming (phase five), were carried out stepping back-and-forth between the steps. For theme generation whiteboards, paper, scissors, and glue and communication with other authors (P.O. and H.Q.), as well as other researchers acting as critical friends were used (Costa and Kallick 1993, Baskerville and Goldblatt 2009). Where needed, to define codes and themes, the researcher went back to the individual transcripts to refine and combine codes. Using the codes and generated themes, the analysis was written up (phase six).

### Reflexivity statement

The lead researcher (C.B.) held her own positive views of parkrun, was of the same gender, albeit younger than the participants and participated regularly in parkruns herself. As a health researcher, she held views on behaviour change, believing that it is possible to change behaviour and having experienced this herself over the course of her life. She regularly participates in parkrun herself as a runner/walker and volunteer. She did not have any negative parkrun experiences, though was aware they exist. Familiarity with parkrun allowed her to connect with the participants and understand specific language used occasionally (e.g. tail walker, run director, or ‘vollie’ for volunteer). Given her experience and knowledge of parkrun, it would have been impossible for her to remove her experience from the data, which reflexive thematic analysis allows for. How the lead researcher’s own parkrun experience impacts the analysis was discussed with the other authors and other researchers acting as critical friends (Costa and Kallick 1993, Baskerville and Goldblatt 2009). C.B. engaged in discussion with the coauthors throughout the data collection and analysis. S.H. is the director of the parkrun Research Boards, with A.B. and H.Q. being co-vice chairs of the research board. All three have conducted previous parkrun research. P.O. is a researcher in a centre that relates to sport participation and has a positive view of the benefits of physical activity. S.H. and H.Q. are active parkrun participants, while A.B. is an infrequent parkrun participant. P.O. has never participated in parkrun but engages in physical activity regularly.

## Results

**Figure 1:**
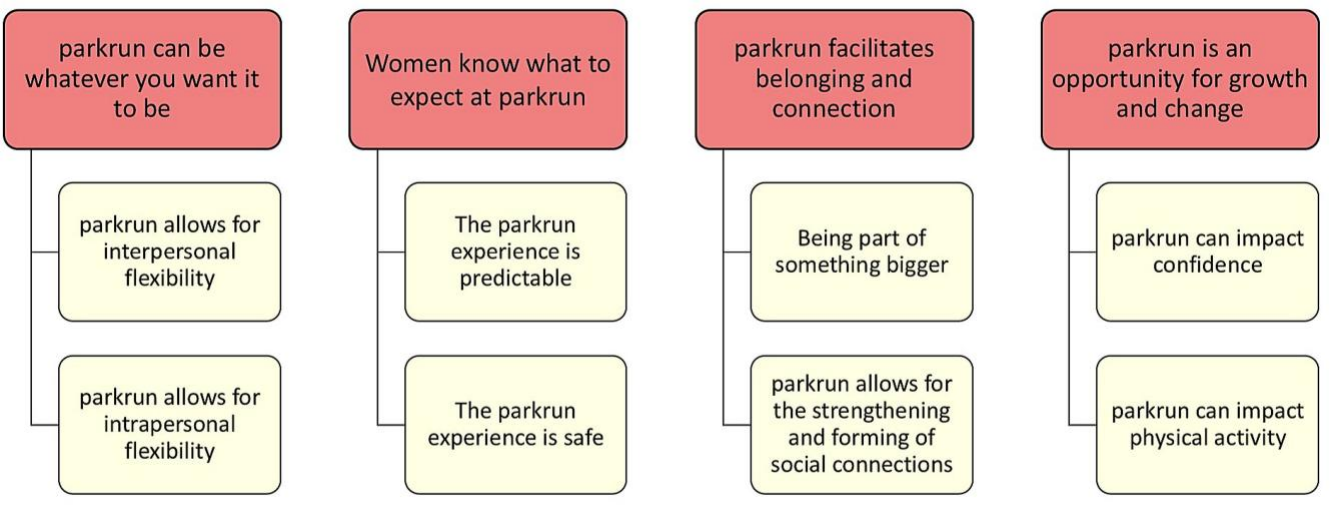
Overview of themes and subthemes.

Four themes, each with two subthemes, were developed to understand what elements of parkrun can be taken forward to other community-based PA to encourage PA among women. An overview of the themes and subthemes can be seen in Fig. 1 and Supplementary Table S3 (with example quotes).

### Theme 1: parkrun can be whatever you want it to be

Theme 1 demonstrates that parkrun participation and the parkrun experience is flexible and adjustable by participants despite set elements, such as the course length or start time. This allows for *interpersonal flexibility* and *intrapersonal flexibility* which shape parkrun engagement.

#### Subtheme 1: parkrun allows for interpersonal flexibility

Participants had a wide array of reasons for participation: an opportunity to be active, part of wider training, seeking a new way to be active, complete challenges, such as the parkrun alphabet (participating in a parkrun event starting with every letter of the alphabet), to be active in a social setting or for the feedback after participating in the form of performance statistics (e.g. their finish time or age-adjusted grading). The social situation at parkrun is also flexible, where some participants appreciated being able to attend with family, while a few spoke about parkrun being their time away from family to be by themselves. Participants commonly stressed that parkrun is not just a run, but can be a walk, or a run-walk combination, and therefore different fitness levels can participate: ‘People of all ages and shapes and sizes come together to enjoy being in the park on a Saturday morning and […] it is totally fine to walk the whole way. Doesn't matter how slow you are or how fast you are’. (Participant 29, 48 years).

Differences expressed by participants included: while some attended for social reasons: ‘The social side's been […] more important than the physical activity, they go hand in hand’. (Participant 11, 42 years) others preferred it as a place to switch off: ‘I might have a quick chat if I see someone that I know, but I'm also happy just to not actually really engage too much’. (Participant 22, 42 years). Some participants had standing arrangements, such as childcare to allow attendance, while others attended when it suited their schedule: ‘parkrun is something that I will go to if I can make it’. (Participant 15, 49 years). A few participants saw parkrun as an enjoyable competitive environment: ‘My parkrun journey is probably more competitive than your usual parkunners’. I would say I… I do come first female and I've done that maybe up to 200 times now at my local parkrun’. (Participant 28, 43 years), whereas others did not care about the competition element, or only competed with themselves: ‘I like getting my result […] I'm actually not really competitive, but it's nice to get because it's just for yourself’. (Participant 7, 49 years). The flexibility of parkrun allows for a wide array of different reasons and preferences of participation in the same community-based event.

#### Subtheme 2: parkrun allows for intrapersonal flexibility

Participants spoke about the flexibility within parkrun, such as choosing how to participate each week: ‘It's really just about on the day… Do you wanna talk during a run? Do you wanna do a [volunteer] job?’ (Participant 22, 42 years). This included being able to attend different locations, whether for fun or when travelling. Flexibility in location allowed the small minority to change event if they had a negative experience at a specific location: ‘I just found [specific location] cliquey […]. Like every other parkrun I've been to, people will start talking to you like ‘where you're from?’ You know, ‘what's your race shirt you're wearing?’ […] it's just as such a different feeling when you go to one that people are talking to you, smiling and yeah, just give you a different feel’. (Participant 14, 46 years).

A common theme was parkrun accommodating for people’s changing preferences and motivations, even weekly. One participant spoke about how she started attending parkrun as speed training but now attends because it allows her to see her friends, suggesting a long-term change in motive. Volunteering, when running/walking are not possible because of injury, was also occasionally discussed regarding a short-term change in participation habits: ‘I started with parkrun and then I injured myself and I couldn't run… I volunteered’. (Participant 3, 41 years). While participants spoke about how life changes such as pregnancy and post-partum impacted their PA preferences and engagement this was not specifically related to parkrun.

Theme 1 describes the flexibility participants describe as part of their parkrun experience and how the parkrun experience can differ between individuals and over time, highlighting its potential as a form of PA for women.

### Theme 2: the parkrun experience is consistent

Another theme constructed was that women experience parkrun to be consistent due to its reliability, as it functions the same at every location, every week. This theme is split into two subthemes: parkrun is predictable and parkrun is safe.

#### Subtheme 1: parkrun is predictable

The majority of women described parkrun as offering the same positive experience every week with steady characteristics: accessible, achievable and available. A few participants discussed how their children’s sport kept them from participating but they knew they could return: ‘I can’t do parkrun so at the moment […] it is clashing with my son's basketball, which will finish soon. So then I'll be able to go back to parkrun’. (Participant 22, 42 years). The predictability helped overcome barriers experienced at initial attendance such as being unsure how it functions, where to park, or whether they would be able to find it. Sometimes these barriers returned if participants attended a new location but parkrun’s consistency helped: ‘I remember trying a new location, it was so silly ‘cause I've done the one at [location 1], but then when I went to [location 2] I still had to go… I'm gonna get lost because it was a different course’. (Participant 7, 49 years). Many participants expressed that parkrun being predictable helped overcome fears of being last, getting lost, or being unable to complete it. Often this was mentioned in combination with the tailwalker (the volunteer who is the last to cross the finish line): ‘I was still a competitive person and she [participant’s sister] just went, you know, you won't come last [because of the tailwalker] and she really sold that you won't come last thing’. (Participant 33, 49 years). Additionally, many participants spoke about the consistent start time and location allows planning and eases attendance. Parkrun comes with a predictability allowing women to know what to expect ahead of time, which may lead to feelings of reassurance and competency and therefore promotes attendance and creates a smooth experience for those attending.

#### Subtheme 2: parkrun is safe

The majority of participants spoke about experiencing psychological and physical safeness, suggesting that parkrun is something that is not dangerous and unlikely to cause harm. The nonjudgmental environment meant they felt allowed to participate in parkrun even if they did not perceive themselves as an active person, runner or walker, due to the absence of external pressure or any need to be competitive: ‘It doesn't have to be competitive. Everyone is pretty friendly.[…] feel like I can't run 5 K, I don't look like an idiot […]. If you’re worried about getting a crappy time, no one, no one really knows. It doesn't matter’. (Participant 12, 45 years). The experience of not feeling watched or judged led to feeling less self-conscious at parkrun, especially compared to experiences in other PA settings, such as gyms. Additionally, participants expressed that it did not matter what they wore or looked like: ‘I think that it works with women around my age […] because when you go to a gym, it's a lot of show off […] but [parkrun] is more like genuine. So you are there to really exercise. You are not there to meet people, to look for a husband or to show off new clothes or anything’. (Participant 10, 48 years). The physical safeness ensures that participants can enjoy exercising outdoors, which they did not always feel safe to do. The presence of other runners and volunteers added safety: ‘You've got marshals around if you know you fall over or whatever, there's people. People will stop and ask if you're OK if something happens’. (Participant 14, 46 years). Women perceived parkrun to be a predictable and safe environment, easing attendance and engagement with parkrun. This highlights that while the type of participation and reasons might change over time (see theme 1), parkrun itself does not change; both of these elements are important to individuals’ parkrun experiences and promote participation.

### Theme 3: parkrun creates connections

For almost all participants, there was a social element to parkrun participation leading to the development of theme 3. This theme has two subthemes: being part of something bigger and parkrun builds social connections.

#### Subtheme 1: being part of something bigger

Occasionally, participants described feeling part of something bigger when attending parkrun in relation to them being in a community that cares about them: ‘I wasn't able to run and there was someone that came up and went ‘I saw you walking today, is everything okay?’ And so… you know, just the fact that people, they didn't necessarily know my name, but they knew me…’ (Participant 24, 45 years). This often led to women feeling passionate about parkrun, wanting others to know about parkrun and to participate, hoping it would lead to them also experiencing these feelings of belonging and connection. A few participants spoke about how they would miss parkrun if it was not there, and missed parkrun, when it was not happening during the COVID-19 pandemic. Some participants felt like they are part of something bigger, which shapes participation and creates a sense of belonging and connection with the community they are active with.

#### Subtheme 2: parkrun builds social bonds

Participants spoke about their social networks and connections in relation to parkrun. This included the deepening of already existing relationships with friends or family members: ‘It's been really nice to have something to connect with both the kids. So having something regular that you know, we go along and often you know […] I wouldn't talk to him [participant’s son] about during the week you know, we'd be so focused on school and homework and so it was an opportunity to spend time together outside of that, and to kind of talk about other things and connect on that level’. (Participant 15, 49 years). Parkrun was also an opportunity to make new connections, with participants attending because someone had encouraged them to. Some connections were superficial, only regarding parkrun, while others went beyond parkrun and led to going on holiday and attending weddings together: ‘And then go for breakfast afterwards with the regulars and […] you do have a separate community I think ‘cause of parkrun’. (Participant 25, 37 years). The social aspect of parkrun brought accountability for some participants. Checking in with friends or family members Friday evening about parkrun the following morning brought a commitment making them more likely to attend. Overall, this theme describes that parkrun is a place where women experience connection and belonging, fostering an enjoyable experience which positively impacts their parkrun attendance. The social connections made and strengthened play a role in their engagement of this community-based PA initiative.

### Theme 4: parkrun allows growth and change

The fourth theme developed describes how participants often experience parkrun allowing growth and change, and how this is important for attendance. This theme consists of two subthemes: parkrun can impact confidence and parkrun can impact physical activity.

#### Subtheme 1: parkrun can impact confidence

This subtheme includes the experience of having grown in confidence by attending parkrun; through learning new skills and enhancing existing ones, such as public speaking: ‘With them asking me to be an RD [run director, person in charge of the event that day] was like I have to get up and talk in front of people […] it definitely has built my confidence in speaking to in front of people’. (Participant 3, 41 years). A small number of participants also spoke about how they did not only experience this in themselves but also saw this happening in others. parkrun gave participants the confidence to exercise; to exercise in public and to exercise beyond parkrun: ‘I think it has [given me confidence] and you know, like you would… I would have been hesitant to go for a run because I'm a slow runner in my earlier days, but […] parkrun, it definitely gives you that confidence’. (Participant 34, 48 years).

#### Subtheme 2: parkrun can impact physical activity

Participants spoke about how parkrun impacted their PA, within and beyond parkrun. For a minority of participants, parkrun was their initial form of organized running or exercise. It had encouraged other forms of PA and led to an overall increase. This was sometimes closely linked to parkrun, such as being active with someone from parkrun or joining a running group after seeing it represented at parkrun: ‘I think without parkrun I would have seen that [joining a running club] as being out of my league like I would have thought that you had to be a really full-on, serious athlete’. (Participant 15, 49 years). Most participants described their perspective on running or PA had changed due to parkrun. For some this was linked to seeing other people at parkrun of a similar body size or shape and age participating and achieving things at or beyond parkrun; for others, seeing other people being active gave them a feeling of permission to be active even if they did not feel like they were very good at it. Overall, parkrun provides an opportunity for women for change and growth in confidence and PA.

## Discussion

Using parkrun as an example, four themes were developed from the interviews giving insight into how community-based PA initiatives might be utilised to increase women’s PA levels: (1) parkrun can be whatever you want it to be, (2) the parkrun experience is consistent, (3) parkrun creates connections and (4) parkrun allows for growth and change. These themes reflect different elements of women’s parkrun experience, and what they found to be unique about parkrun compared to other PA, highlighting why parkrun works well and what can be learned from it. Recommendations for community-based initiatives developed from the findings are displayed in Table 1.

**Table 1:** Summary of recommendations, their rationale and potential actions based on the findings.

Recommendation	Rationale	Potential actions
Community-based initiatives should emphasise regularity and repetition.	Weekly occurrence at the same place, at the same time creates safeness, allows for habit building and creates a predictable experience, helping women to overcome barriers associated with PA and attendance.	Initiatives that happen at the same time, at the same place, weekly (or another regular time sequence) Initiatives that follow a similar pattern every time they occur
Community-based initiatives should have flexibility in attendance.	While regularity and repetition are important, flexibility for attendees might be of benefit. Being able to attend an initiative when possible but there being no consequences if someone is unable to attend once or over a longer period (such as due to children’s sporting commitments) might encourage overall attendance. Flexibility in the type of participation also might be of benefit. Women appreciated being able to move from running to walking or volunteering, depending on the day or their current life circumstances (such as injuries).	Allow to pause attendance of activity without consequences Make it easy to resume activity after a break Have flexibility in the engagement in activities (e.g. different levels of difficulty that can be changed weekly) Allow for continued engagement if injured or unable to participate in activity for different reasons
Community-based initiatives should have elements of social engagement and autonomy over the level of engagement.	The ability to connect with others, whether with people they already know or making new connections, is of benefit. It can also create accountability and increases the desire to engage in the activity, as it is not just for the sake of the activity but rather goes beyond that. At the same time, there is no pressure to interact beyond the interactions necessary for the activity.	Create low pressure opportunities for social engagements during and afterwards activity (e.g. interaction with other participants during/immediately after, opportunity to meet for coffee afterwards)

### Health promotion theories

In addition to the wider literature, the findings are discussed in the context of health promotion and behaviour change theories. Community-based initiatives are commonly based on socio-ecological models, proposing that behaviour is not solely the result of an individual’s knowledge, values and attitudes but also by social influences, the organization and the wider community they engage with (McLeroy *et al*. 2003), originating in the work of Bronfenbrenner (Bronfenbrenner 1979). Socio-ecological models of PA often include five levels: intrapersonal (within the person, focusing on biological and psychological concepts), interpersonal/cultural (e.g. family members, friends, neighbours and contacts at work), organizational (effects of personal relationships or organization characteristics), physical environment/community (including the social and physical environments) and policy (e.g. laws and regulations) (Sallis *et al*. 2015). For the interpretation of the findings of this study, the intrapersonal level is broken down by the COM-B model, which is part of the behaviour change wheel framework (Michie *et al*. 2011). The COM-B model (Michie *et al*. 2011) states that the combination of capability, opportunity and motivation increase the probability of a behaviour, in this case participating in parkrun. Capability is further broken down into physical and psychological, motivation into reflective and automatic, and opportunity into physical and social. Additionally, where relevant, self-determination theory is included which proposes three basic human needs are related to motivation: competence, the sense of confidence individuals have towards the action; relatedness, the sense of connectedness they feel with other people; and autonomy, the feeling of the behaviour being an expression of their own desires and wants (Ryan and Deci 2000, Lloyd and Little 2010).

**Figure 2:**
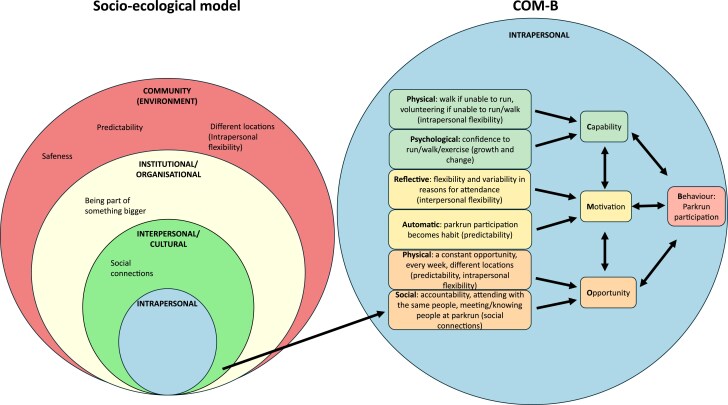
Findings mapped on to the socio-ecological model and COM-B.

Figure 2 shows how the findings map onto the socio-ecological model and COM-B. The relevance of self-determination theory is highlighted throughout the text.

### Parkrun can be whatever you want it to be

Though previous parkrun literature has not classified the flexibility of parkrun as inter- and intrapersonal, similar elements have been discussed. Research in the UK highlighted parkrun’s accessibility and inclusivity, as the simple set-up and free attendance allows people with different preferences and needs to attend (Stevinson *et al*. 2015). This is connected to interpersonal flexibility, showing that parkrun caters for a variety of preferences and needs. One example highlighted in the interviews was the possibility to attend parkrun with children and family, which might be especially relevant for women as family duties and childcare responsibilities are a commonly expressed barrier for women engaging in PA (Peng *et al*. 2023), and the opportunity to attend parkrun with family and children might help overcome this. Intrapersonal flexibility has been emphasized in previous parkrun research, highlighting the choice of being able to participate or not, which location to attend, and the variety of volunteer roles for participants with mental health difficulties (Morris and Scott 2019). Other research has found there is a shift in the parkrun experience over time in interviews with men (Dunne *et al*. 2024) and the fluidity of the parkrun experience allows to move in and out of parkrun, between events, social groups, running, walking and volunteering (Cranney *et al*. 2025). This study adds that parkrun can accommodate shifts over time in women’s lives, both short-term (e.g. injury) and long-term (e.g. reasons for exercise) activity preferences and changes. Research has shown that PA participation changes over the course of a lifetime (Gropper *et al*. 2020), and this may be because women undergo many physical and role changes throughout their lives (McArthur *et al*. 2014), which was mentioned in the interviews as influencing overall PA but not specifically relating to parkrun. Parkrun’s ability to be whatever individuals want it to be keeps it a relevant form of PA when life circumstances or motivations for activity change; this flexibility in attendance seems to be an important element of parkrun participation. Community-based initiatives should consider elements of flexibility in participation to increase women’s PA levels.

Previous research has found women were more intrinsically motivated to be active and enjoyed the activity more when it was perceived to involve choices (Henderson and Ainsworth 2003, Lloyd and Little 2010), which the flexibility of parkrun might provide. This could be interpreted as participants having autonomy over their experience, an element of self-determination theory (Ryan and Deci 2000). The flexibility could also give participants a sense of agency, meaning they act out of personal interest and integrated value, another element of autonomy. Parkrun's ability to be whatever you want it to be might therefore increase participation and motivation for participation. The flexibility of parkrun gives participants the psychological capability (a COM-B component), by walking if unable to run or volunteering if injured or wanting to walk/run can be linked to psychological capability. The variety of reasons suggests reflective motivation drives attendance (another COM-B component). Being able to attend different locations is part of the community/environment of the socio-ecological model, and leads to an increase in engagement. This suggests that community-based PA initiatives should have elements of flexibility that allow for different forms of participation and a variety of different reasons for attendance (e.g. competition, fitness, or social reasons).

### The parkrun experience is consistent

The subtheme of parkrun being a safe experience has been reported in previous literature, such as parkrun being a safe environment to be active in (Cleland *et al*. 2019) including for individuals with long-term and mental health conditions (Morris and Scott 2019, Quirk and Haake 2019). Safety concerns are a commonly reported barrier of PA for women (Peng *et al*. 2023), and women’s experiences of exercising outdoors are influenced by fear and risk (Roper 2016). Parkrun providing a safe environment for women to be active in might increase participation and enjoyment of exercise as participants do not experience fear while exercising. This highlights the importance of creating a safe environment for community-based PA initiatives that aim to increase women’s PA levels.

The weekly occurrence of parkrun and it functioning identical in all locations makes it different to other mass-participation events by providing a consistent experience which allows for habit building: a relationship between habit building and PA has been found, where once the habit is established, participants are more likely to engage in the behaviour (Feil *et al*. 2021). Parkrun’s predictability allows for habit building and therefore encourages regular attendance and increased PA. One barrier women commonly experience to PA is childcare responsibilities (Peng *et al*. 2023). Within this theme, the participants described having to take a break from parkrun due to time-conflicts with their children’s sporting activities but valued the predictability and consistency of parkrun allowing them to return easily when the time allowed. This underscores the importance of community-based initiatives being regular and consistent, supporting habit formation and ensuring continuity.

Parkrun providing an environment where participants feel comfortable based on the safeness and predictability provided shows that it created a physical opportunity to be active in, a component of the COM-B model. Some women mentioned they valued the opportunity to be active outside, which can be linked to the community/environment layer of the socioecological model. The predictability can be connected to automatic motivation, part of COM-B, where participants have built the habit of parkrun participation. Parkrun’s predictability is also an important part of its environment/community, one of the layers of the socio-ecological model and might influence behaviour through this. Overall, this theme suggests that for women an important part of community-based initiatives is the weekly occurrence in the same place, showing that community initiatives should have an element of regularity and repetition.

### Parkrun creates connections

The subtheme being part of something bigger and this increasing participants’ commitment to parkrun is in line with previous research that higher levels of identification with parkrun increase participation (Stevens *et al*. 2019) and that participants have an emotional connection to their local parkrun, the larger movement, or both (Bowness *et al*. 2022). Women often seek meaningful exercise (McArthur *et al*. 2014), which might be met through them experiencing being part of something bigger at parkrun, leading to increases in enjoyment and parkrun engagement. Previous research has highlighted the opportunity parkrun brings for social connection: parkrun creates a sense of belonging, it is more than just being active in a park and the social cohesion of parkrun facilitates continued involvement (Hindley 2020). This fits with the other subtheme of Theme 3, of participants making social connections, which lead to accountability and therefore increase the likelihood of them attending parkrun, as previous research has suggested that social accountability may increase PA (Forster *et al*. 2020). Additionally, it has been found that parkrun participation often occurs through existing personal contacts (Wiltshire and Stevinson 2018), and families often play a role in initiating parkrun participation (Bowness *et al*. 2022). This theme adds that parkrun also allows the strengthening of existing relationships with families and friends. Lack of social support is a common barrier of women for PA (Peng *et al*. 2023) and the social connections made at parkrun might provide encouragement and social support that increase PA adherence. This suggests that community-based initiatives should include opportunities for social engagement.

The social setting of parkrun influences participation through creating new connections, strengthening existing connections, accountability and creating meaningful experiences by participants perceiving themselves to be part of something bigger. This is in line with parkrun providing a social opportunity, a COM-B component, and an individual’s social and physical environment influencing behaviour, as part of the socio-ecological model. Additionally, participants feel part of parkrun (‘being part of something bigger’), showing that the organization/institutional level influences participation. This theme also suggests that parkrun promotes relatedness, a component of self-determination theory (Ryan and Deci 2000), and this sense of connectedness at parkrun might lead to increases in wellbeing and motivation. Including social engagement elements in community-based PA initiatives might be important to increase women’s participation in them.

### Parkrun is an opportunity for growth and change

Previous research has found parkrun participation leading to an increase in PA and confidence for activity (Stevinson and Hickson 2018) and running specific confidence (Stevens *et al*. 2019), as well as participants reporting joining running groups as a result of parkrun participation (Bowness *et al*. 2020). Theme 4 suggests that these elements are connected: women experience an increase in confidence through parkrun participation, which leads to them feeling encouraged and emboldened to partake in further activity and to a mindset shift regarding being active. Additionally, for some participants growth and change are not just linked to running or PA but also volunteering at parkrun and the confidence and skills gained from it. Therefore, beyond changing women’s opinions on exercise, parkrun can also impact their wider lives. Confidence is often present in behaviour change theories, which could explain why an increase in it leads to more PA participation and parkrun being an opportunity for growth and change. parkrun participation might promote self-efficacy, the belief in one’s own abilities. Confidence/self-efficacy is a well-established determinant of PA, suggesting that if an individual displays higher levels of confidence and self-efficacy, they engage in more PA (Bauman *et al*. 2012). The increase in confidence can also be linked to an increase in psychological capability of the COM-B model (Michie *et al*. 2011). Experiencing feeling competent at parkrun, through the confidence gained, might lead to an increase in feeling capable, which promotes motivation and behavioural change, i.e. an increase in PA.

### Summary of findings and recommendations

While Themes 1 and 2, the flexibility and consistency of parkrun, may initially seem contradictory, they complement one another: because parkrun is consistent, and women know what to expect, it has a predictable flexibility where participants know they can change their participation type or location depending on the week or life season they are currently in. Parkrun being whatever participants want it to be might also be what allows for belonging and connection, as well as growth and change, themes in which participants shared different experiences about the social environment at parkrun and the impact of parkrun on the rest of their lives. Some of the themes, especially 1 and 2, highlight what is important to women about their parkrun experience and might give insight into parkrun’s successful engagement of women in their initiative, and therefore elements that should be considered when designing community-based PA initiatives for women. Themes 3 and 4 highlight especially how elements of the parkrun experience go beyond parkrun and explore how parkrun’s impact goes beyond just exercise on a Saturday morning. This gives insight into the potential of community-based initiatives and how they can impact women’s lives. Table 1 summarises the recommendations for community-based initiatives based on the findings. This includes recommendations for regularity and repetition, flexibility in attendance and elements of social engagement.

### Limitations

While some demographic information was known about the participants and indicated a good diversity regarding parents’ birth countries and some variation regarding language spoken at home, no participants were Aboriginal or Torres Strait Islander, and less participants were from disadvantaged backgrounds. One limitation of the research is that no attention was paid how demographic variables influence parkrun participation (such as ethnicity, disadvantage level or body composition). Future research should take other potential barriers such as disadvantage levels or health conditions into consideration, as some demographics would exacerbate the risk factors for physical inactivity further, and research has shown that people from deprived communities are less likely to participate in parkrun (Smith *et al*. 2021). This suggests that when aiming to engage participants from more disadvantaged background, other elements not considered in this study might be important. Another limitation was all participants had previously completed a parkrun survey. While this eased recruitment, only people who could participate were contacted, and this survey had been distributed to all parkrun participants in Australia, this might have introduced bias to the sample, such as availability bias and selection bias (Centre for Evidence-Based Medicine 2025). This method also might limit the transferability to all female parkrunners, as likely individuals who are more engaged in parkrun responded to the survey, left their email address, and replied to the recruitment email about the interviews. Future research should consider recruiting differently, by including individuals who have not participated in previous research, in order to capture a wider perspective on parkrun and its impact. As some of the findings align with previous literature, such as parkrun’s accessibility and inclusivity allowing people with different preferences and needs to attend (Stevinson *et al*. 2015), it is likely that some of these findings extend beyond women in Australia aged 35–54 and can be expanded to women and participants in other countries. However, cultural and contextual factors can impact PA behaviours and beliefs (Rio and Saligan 2023), which might limit the generalisability of the results.

## Conclusion

The women interviewed valued parkrun for its ability to adapt to their current and long-term needs within the set parameters of parkrun that provide predictability and safeness. The social elements of parkrun allow the experience to be about more than just being active. The benefits of parkrun also go beyond parkrun, including gaining confidence for and beyond PA, and paving participants’ paths to more PA. Considering elements of regularity and repetition, allowing for flexibility in attendance and creating room for social engagement may be of importance when planning PA initiatives that aim to increase the activity levels of women.

## Supplementary Material

daag081_Supplementary_Data

## Data Availability

The data underlying this article cannot be shared publicly to protect the privacy of participants. An anonymised version of the data will be shared on reasonable request to the corresponding author.
