# Supplementary material for: Exploring parkrun experiences of women aged 35 to 54 in Australia: a qualitative study
Source: Health Promot Int. 2026 Jun 16;41(3):daag081. doi: 10.1093/heapro/daag081 (PMC13271247; doi:10.1093/heapro/daag081)
Supplement: daag081_Supplementary_Data [file daag081_supplementary_data.zip › Table S1.docx]

Table S1: Interview guide

|  | _Find out_ | _Question/Prompts_ |
| --- | --- | --- |
| _Introduction_ | _How long they have been registered with parkrun_  _How regularly they attend parkrun_  _How they found out about parkrun_  _Why they came along to parkrun_ | _Could you tell me about your parkrun experience?  How did you find out about parkrun?_  _How did you experience your first parkrun?_  _What made you come back?_  _Could you tell me about your typical weekly physical activity?_  _What other physical activity events have you participated in?_ |
| _(Past) Physical activity_  _Physical activity journey_ | _What their physical activity was like before parkrun_  _What their physical activity is like now_  _What other physical activity they do besides parkrun_  _What other physical activity events besides parkrun they have participated in/do participate in_ | _Thinking back to when you first registered with parkrun…_  _What was your physical activity like before you registered for parkrun? What kind of things were you doing and how often?/ Tell me about what your physical activity was like when you registered with parkrun/started attending parkrun._  _Talk me through your relationship with physical activity over time._  _Do you like physical activity?_  _Is it something you’ve always liked?_  _Has parkrun made any difference to your physical activity?_  _What do you mean by [what they just mentioned]?_ |
| _Changes in physical activity through parkrun_ | _If and how parkrun has changed and affected their physical activity_ | _Has parkrun changed your physical activity/your relationship with physical activity?_ |
| _Effect of parkrun on physical activity_ | _Why has parkrun had an impact on their physical activity_  _What element parkrun had an impact on_  _If needed, specific elements parkrun might have ad impact on_ | _How do you feel thinking about physical activity?_  _What do you think it is about parkrun that has changed your physical activity?_  _What is important to you about your parkrun experience?_  _Has parkrun changed the reasons for which you do physical activity?_  _Is participating in parkrun different from other physical activity you do?_  _If more specific questions are needed: Has parkrun had an impact on … your confidence/motivation... in what ways?_  _Think about competition element (big gender differences)_  _Think about non-judgemental/physically safe environment_  _Ever felt excluded at parkrun/impact of that?_ |
| _Other_ |  | _Have other people had an impact on your parkrun experience?_ |
