# Supplementary material for: Exploring parkrun experiences of women aged 35 to 54 in Australia: a qualitative study
Source: Health Promot Int. 2026 Jun 16;41(3):daag081. doi: 10.1093/heapro/daag081 (PMC13271247; doi:10.1093/heapro/daag081)
Supplement: daag081_Supplementary_Data [file daag081_supplementary_data.zip › Table S2.docx]

Table S2. Counts of female parkrun participants in Australia by age group.

| Age Category | Count Female Participants | Percentage Female participants |  | Age Category | Percentage Female participants |
| --- | --- | --- | --- | --- | --- |
| 16-19 | 26959 | 7.02 |  | 16-34 | 33.04 |
| 20-24 | 24744 | 6.44 |  | 35-54 | 50.70 |
| 25-29 | 31339 | 8.16 |  | 54+ | 16.26 |
| 30-34 | 43872 | 11.42 |  |  |  |
| 35-39 | 51555 | 13.42 |  |  |  |
| 40-44 | 53736 | 13.99 |  |  |  |
| 45-49 | 49326 | 12.84 |  |  |  |
| 50-54 | 40170 | 10.46 |  |  |  |
| 55-59 | 25700 | 6.69 |  |  |  |
| 60-64 | 17474 | 4.55 |  |  |  |
| 65-69 | 10429 | 2.71 |  |  |  |
| 70+ | 8859 | 2.31 |  |  |  |
| Total | 384163 |  |  |  |  |
