# Supplementary material for: Exploring parkrun experiences of women aged 35 to 54 in Australia: a qualitative study
Source: Health Promot Int. 2026 Jun 16;41(3):daag081. doi: 10.1093/heapro/daag081 (PMC13271247; doi:10.1093/heapro/daag081)
Supplement: daag081_Supplementary_Data [file daag081_supplementary_data.zip › Table S3.docx]

Table S3. Themes and subthemes with quote examples.

| Theme | Subthemes | Quote |
| --- | --- | --- |
| parkrun can be whatever you want it to be | parkrun allows for interpersonal flexibility | “Everyone's just there... I guess doing their own thing.” Participant 8, 46 years |
|  | parkrun allows for intrapersonal flexibility | “It [approach to parkrun] has changed simply for the fact that I'm a lot slower than I was, so I have to accept that, and I really like volunteering. I have volunteered in, in dribs and drabs over the few years that I was beginning. But I was more about I had to turn up and run because it was my speed work or I need that because the next event is coming.” Participant 19, 55 years |
| The parkrun experience is consistent | parkrun is predictable | “I trained rurally, and we have to move around like in my third year I think I moved something like 20 or 30 times for placements and parkrun is just so good because nearly every single town that we're placed in has one, and you can do it…” Participant 6, 39 years |
|  | parkrun is safe | “Like as women, I don't walk through naturally secluded parks by myself. I know other people do, but some of those paths are quite secluded. I just don't. So I think if you wanted to do that, it's [parkrun] quite it's safe because there's people around. Also, if you weren't sure if you could make it, you've always got like marshal, marshals or the tailwalker. So if something was to happen to you, you're not gonna just get forgotten about or missed or whatever, and they'll be somebody to sort of look out for you.” Participant 7, 49 years |
| parkrun creates connections | Being part of something bigger | “So, for me, doing parkrun, it's my social event. And I love seeing the regulars. I love volunteering for it. I love to be out doing it.” Participant 19, 55 years |
|  | Parkrun builds social bonds | “and another big reason that I also went back was because my father who also lives locally, he has like a quite a serious mental health episode and in order to encourage him to um, start exercise again and to get out in the community I asked him if he would come with me.” Participant 22, 42 years |
| parkrun allows for growth and change | parkrun can impact confidence | “I think it's [parkrun] given me the ability to be able to go to wherever I like to... I like to do every now and then a tourist once. I'll go to a different place um with I wouldn't have had the confidence to do that, you know, 10 years ago or whatever, but now I'll just roll up, find out where they start, have a bit of a chit chat to people and run their course and thank them afterwards.” Participant 14, 46 years |
|  | parkrun can impact physical activity | “If I hadn't been going to parkrun um, I wouldn't have actually probably become like a social runner at all. I wouldn't I probably won't be doing anything. I don't think I'd be doing any physical activity.” Participant 25, 37 years |
