# Supplementary figures and images for: Exploring parkrun experiences of women aged 35 to 54 in Australia: a qualitative study
Source: Health Promot Int. 2026 Jun 16;41(3):daag081. doi: 10.1093/heapro/daag081 (PMC13271247; doi:10.1093/heapro/daag081)

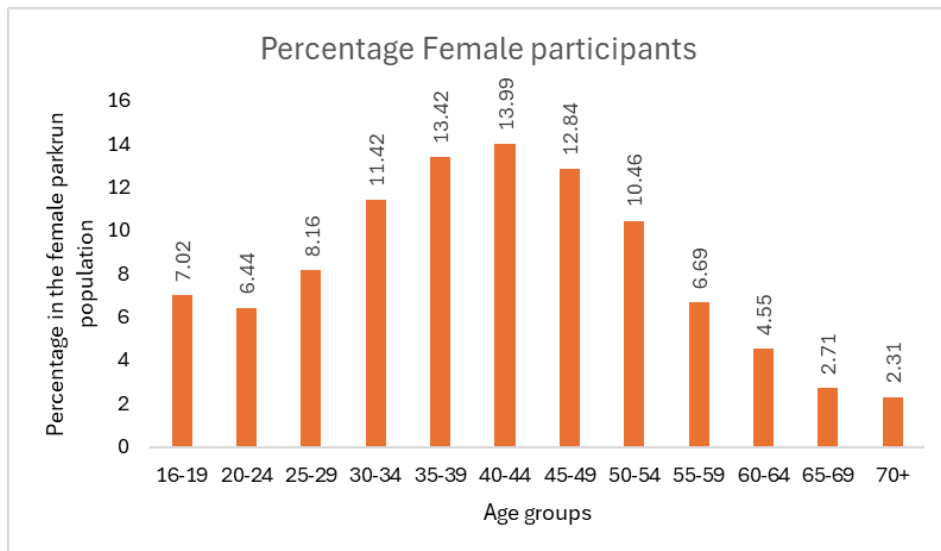

Figure S1. Prevalence of female parkrun participants by age group.

Supplement: daag081_Supplementary_Data [file daag081_supplementary_data.zip › Figure S1.pdf]

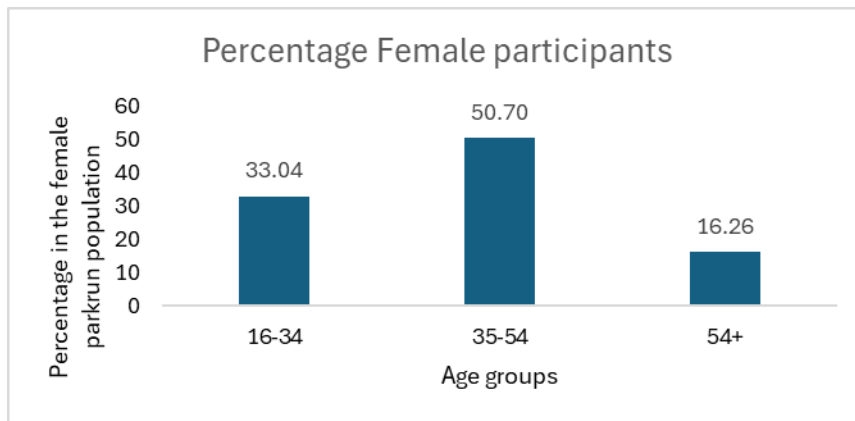

Figure S2. Percentage of female participants in Australia by age group comparing 35-54 to 16-34 and 54+.

Supplement: daag081_Supplementary_Data [file daag081_supplementary_data.zip › Figure S2.pdf]
